# The Discomfort of Death Counts: Mourning through the Distorted Lens of Reported COVID-19 Death Data

**DOI:** 10.1016/j.patter.2020.100066

**Published:** 2020-06-16

**Authors:** Inioluwa Deborah Raji

**Affiliations:** 1 AI Now Institute, New York University, New York, NY, USA

## Abstract

In data science, there’s long been an acknowledgment of the way data can flatten and dehumanize the people they represent. This limitation becomes most obvious when considering the pure inability of such numbers and figures to truly capture the reality of lives lost in this pandemic.

## Main Text

Something about the datafication of lives dehumanizes them.^1^ It is disquieting to imagine these data points as anything other than a compact resource, one in which we as data scientists and dashboard builders feel entitled to package and expose, to harvest and exploit. But data are not bricks to be stacked, oil to be drilled, gold to be mined, opportunities to be harvested. Data are humans to be seen, maybe loved, hopefully taken care of. Data science is human subject research.^2^ When we aggregate, we obfuscate the humanity of those our systems represent and impact, partially because we are actually scared of the human hiding within.

In data science, we try to help people understand with comparisons—measuring and juxtaposing to be able to say with definitive authority that the elephant is larger than the mouse, that the feather is lighter than the stone. No American died in the 2003 SARS or 2014 Ebola outbreak. 12,469 deaths from the 2009 H1N1 swine flu. According to the CDC, 34,200 died from influenza in the last year, down from the 61,000 the previous season—a record high death count from the last 4 decades. (All featured numbers and figures were provided by the Centers for Disease Control and Prevention [CDC].)^3^ This year’s flu season is projected to be somewhere between those figures. With the coronavirus, it is undoubtedly worse. Even with testing overestimated,^4^ death underreported,^5^ and data collection delays of days and weeks—the mortality rate a shrunk proxy of the true deadliness of the disease—this death toll still stands as the worst for a pandemic in the United States since 675,000 perished from the 1918 Spanish flu over a century ago.

Now there are well over 100,000 data points, filtered and screened, to be looked at in amalgamation or sorted into containers of all sorts, to be studied anxiously, to tell me something. Each data point is dead, and dead in the same way data always die. What I am counting are bodies, leeched of life—in this case, probably put to rest alone, likely mourned from afar. In this case, many of the bodies are wrinkled and soft, often Black, calloused from poverty or weakened in some way from pre-existing conditions. These data points now represent those the most at risk of being overlooked and neglected in our society: the elderly, the disabled and sick, Black people, and poor people. Those who had to rely on fervent advocacy for their lives to matter in the first place. In our attempt to better understand them, we package our points with strange labels like “non-Hispanic white,” “non-Hispanic black,” “Hispanic,” and “other race”—the latter encompassing all of Asian, American Indian/Alaskan Native, multiracial, and persons for whom “race/ethnicity data is unknown.” We slot them into differently sized buckets jumping from 0–4 years to 18–49 years. We filter by ZIP code and poverty level. We anchor each death to a location, breaking down regional contributions to map out the city, state, or country to distance ourselves from, and to blame. These deaths are accumulated as points in golf, with an objective of limiting the number of strokes in an attempt to stay under par. We try to highlight these people in our own way, to make them less invisible, but do not succeed in adding just enough dimensions to make them more human.

Not too long ago,^6^ in early April, the president was already so sure we would never get here—decidedly stating that final figures would be “significantly lower” than 100,000, a number he had already pitched as a successful outcome “if you look at what original projections were—2.2 million.” 2.2 million is the predicted death count of letting the deadly virus rage on without any public health interventions whatsoever. The calculated human cost of doing nothing,^7^ including keeping borders open. Bringing in a bigger number, the biggest imagined number, of 2.2 million and putting it beside a very real smaller number approaching 100,000 is done on purpose: to dwarf the severity of reality and imply that things could be worse.

This is what Rudy Giuliani^8^ does when he stacks up the COVID-19 death count to 609,640 cases of cancer, 647,000 Americans dying annually from heart disease, and an estimated 300,000 deaths per year due to the obesity epidemic as he denounces investment in contact tracing. This is what Dr. Phil tries when he talks about how “45,000 people a year die from automobile accidents, 480,000 from cigarettes, 360,000 a year from swimming pools”^9^ to argue for the immediate re-opening of the economy. (Although the smoking and car accident figures approximate the truth, according to the CDC, swimming pool deaths are in fact around 3,500.) The White House itself releases bar graph figures comparing the disease death count to notable war fatalities, including the 498,332 dead bodies from the Civil War, America’s deadliest battle, and the 405,399 gone from World War II.^10^

And in response, we retort with more numbers—better numbers, cleaner numbers, better packaged numbers of more valid comparisons. A death from cancer or a car accident in no way equates the death from a contagious, airborne virus in the midst of a pandemic, we might say. But we’ve already missed the point. It is too easy to reduce a death to a tick mark in a tally to be compared with any other tally of life and death. If we find ourselves simply comparing numbers, something is missing—we have already lost.

In data science, there is the frustrating insistence that our counting is neutral and lacks consequences. That our numbers will protect us and play the critical role in some objective presentation of bigger and smaller, of better and worse. We assume true power lies within the implied stories—these impactful narratives our data will either support or contradict. However, the truest story to be told on these dashboards is the simple fact that someone, somewhere, is forever gone. The most fragile lives are broken, and those most desperately held unto are lost. If we were to approach our death counting with the intentionality of individual mourning, how would we react differently and who would we finally notice? If I could see faces and names on my dashboards, perhaps it would be that much harder to ignore the human hiding and that much easier to understand the weight of meaning that this count holds. Maybe it would be that much more evident that to in any way dismiss or neglect or ignore this count as it rises is to discount and abandon an entire lifetime of personality and purpose. Perhaps it would finally spark sympathy among death counters and database designers to a discomfort too many of us refuse to know.

Because this is how I came to understand the reality of the pandemic’s threat: I have listened as loved ones mourned direct family they could no longer go to visit. From up close and even as far as three degrees of separation away, I can feel it. This person—this living, breathing person once known is now dead, and added to the count I monitor religiously, multiple times a day. I’m not sure yet what we need to do to remember the people whose lives were lost in this pandemic, but I hope it won’t just be through a tally mark on a digital counter. I hope we can begin to find their stories, showcase their lives, and remind people of the fact that as individuals, they mattered.
